# Functionalizing metal nanoclusters in water: Synthesis, interfacing, and emerging applications

**DOI:** 10.1093/pnasnexus/pgaf409

**Published:** 2026-01-02

**Authors:** Yifan Wang, Rukai Zhao, Zhucheng Yang, Jianping Xie

**Affiliations:** Department of Chemical and Biomolecular Engineering, National University of Singapore, Singapore 117585, Singapore; Joint School of National University of Singapore and Tianjin University, International Campus of Tianjin University, Fuzhou 350207, P.R. China; Department of Chemical and Biomolecular Engineering, National University of Singapore, Singapore 117585, Singapore; Joint School of National University of Singapore and Tianjin University, International Campus of Tianjin University, Fuzhou 350207, P.R. China; Department of Chemical and Biomolecular Engineering, National University of Singapore, Singapore 117585, Singapore; Department of Chemical and Biomolecular Engineering, National University of Singapore, Singapore 117585, Singapore; Joint School of National University of Singapore and Tianjin University, International Campus of Tianjin University, Fuzhou 350207, P.R. China

**Keywords:** metal nanoclusters, water solubility, interfacial design, biomedicine application, functional catalysis

## Abstract

Atomically precise metal nanoclusters (MNCs) are metallic kernels wrapped by ligand shells whose chemistry dictates solubility, stability, and interfacial reactivity. Focusing on water-soluble MNCs, this review treats the interface as the bridge from synthesis to function. We map out both direct aqueous syntheses and gentle postsynthetic routes that install hydrophilic motifs while preserving atomic structures. Design rules are then codified: interfacial structure controls stability and mobility, recognition at the interface dictates selectivity, and the local environment sets charge transfer and photophysical responses. Furthermore, ligand shells can define nanoscopic reaction pockets that steer substrate approach and govern electron or energy transfer. These principles illuminate unique advantages in bioimaging and labeling, enzyme-compatible sensing, and controlled charge and mass transport for electrocatalysis. By consolidating synthesis, interfacial physics, and use cases within one framework, this review provides actionable guidelines for linking molecular structure to macroscopic performance and for rationally engineering aqueous MNCs for biomedical and catalytic applications.

## Introduction

Atomically precise metal nanoclusters (MNCs) occupy the size regime between molecular complexes and plasmonic nanoparticles, with structures best described as a metallic kernel enveloped by a protective ligand shell (1–5). Because of their ultrasmall dimensions, the ligand layer constitutes a substantial fraction of the overall structure and thus decisively governs solubility, stability, charge distribution, optical/electronic responses, assembly behavior, and interfacial reactivity (6–8). In this regard, the choice and chemistry of ligands not only determine the structural integrity of the cluster but also govern how MNCs interact with their surrounding environment.

One of the most important illustrations of this principle lie in the solubility of MNCs. The solubility is intrinsically dictated by the chemical nature of the protecting ligands, in which surface polarity, charge state, and hydrogen bonding capability are playing decisively. In traditional organic ligands, apart from the anchoring group (e.g. thiol, alkynyl), the remaining framework is often composed of purely alkyl chains or aromatic hydrocarbons, which renders the clusters insoluble in water but readily crystallizable in nonpolar solvents—an advantage for elucidating precise structures by x-ray–based crystallography, yet at the same time imposing challenges for applications that demand polar or aqueous environments. Water compatibility of MNCs, by contrast, can be obtained when their surfaces are endowed with hydrophilic motifs, such as charged functional groups or biomolecular handles. This can be typically achieved either by direct aqueous synthesis or through postsynthetic modification (9–11). Once dispersed in water, the properties and behaviors of clusters are primarily governed by interfacial chemistry. Hydration shells and counterion atmospheres determine colloidal stability and transport (12). Hydrogen bonding and specific recognition impart selectivity toward biomacromolecules or analytes (13, 14). Moreover, dielectric screening, pH, and ionic strength modulate redox potentials and photophysical responses (15, 16). Finally, the ligand layer can define a nanoscopic reaction pocket that directs substrate approach and regulates electron or energy transfer (17, 18). These interfacial effects highlight the unique advantages of water-soluble MNCs, ranging from reduced nonspecific adsorption in bioimaging and labeling (19, 20), to compatibility with enzyme media in sensing (21, 22), and to controlled charge and mass transport in electrocatalysis and photocatalysis (23–25).

**Fig. 1.**
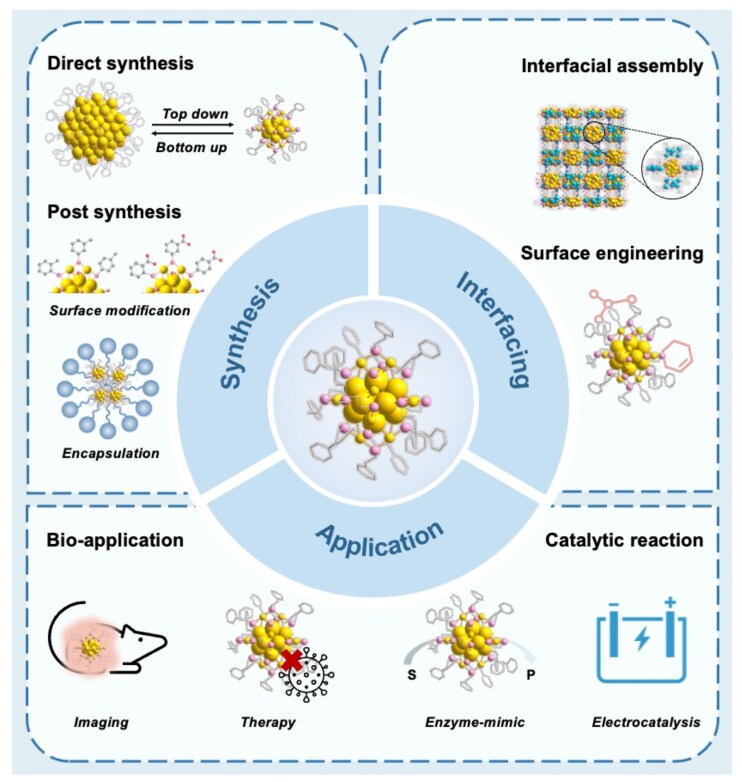
Schematic illustration of synthetic routes to water-soluble MNCs; interfacial programming strategies—including direct surface modification, encapsulation, and assembly—that tailor hydration shells and counterion environments, and emerging applications in bioimaging, therapy, and catalysis.

Prior reviews have surveyed atomically precise MNCs, emphasizing synthesis, structure determination, and optical/catalytic properties, mostly in organic media (8, 26–29). For water-compatible systems, some focused surveys summarize synthetic strategies and bio-related applications (9, 21, 30). Together, these works provided helpful insights for designing and interpreting water-compatible MNCs systems. In this review, we organize recent advances along a coherent chain from (i) synthetic routes that endow MNCs water solubility, to (ii) interfacial design principles that bridge molecular structure and macroscopic functionality, and (iii) emerging use cases in biomedicine and catalysis. By centering on “interfaces” as the connector between synthesis and performance, we aim to provide a practical framework for the rational design of aqueous MNCs (Fig. 1).

## Synthetic strategies for water-soluble MNCs

Achieving water solubility is a key prerequisite for the application of MNCs in biological environments and aqueous-phase catalysis. Various synthetic strategies have been developed to endow MNCs with solubility in water, and these strategies can be broadly categorized based on when water solubility is introduced: either during the synthesis process or through postsynthetic modification. In direct synthesis, hydrophilic ligands such as carboxylates, sulfonates, and ether-containing groups in addition to the anchoring groups are used during cluster formation. These ligands establish hydrogen bonding and electrostatic interactions with water, which confer solubility in aqueous media. Postsynthetic modification relies on adjusting the interfacial interaction between the ligand shell and the solvent, for example by introducing polar functional groups or by encapsulating clusters with hydrophobic ligands in amphiphilic matrices such as polymers, surfactants, or lipid vesicles to achieve stable dispersion in water.

### Direct synthesis

Direct synthesis uses water-soluble ligands to stabilize clusters during formation and can be divided into bottom-up and top-down methods. The bottom-up approach begins with small precursors that grow into clusters, whereas the top-down approach starts from larger nanoparticles (NPs) that are etched by excess ligands to produce small, well-defined clusters. Both routes provide valuable platforms for studying the growth mechanisms of MNCs. For example, in aqueous systems, ligands and intermediates often ionize readily, inherently forming charged species that are directly detectable by mass spectrometry (MS), enabling real-time tracking of NC evolution (31, 32). Other in situ techniques such as nuclear magnetic resonance (33) and ultraviolet-visible spectroscopy (UV-vis) (34, 35) have also contributed important mechanistic insights.

#### Bottom-up method

**Fig. 2.**
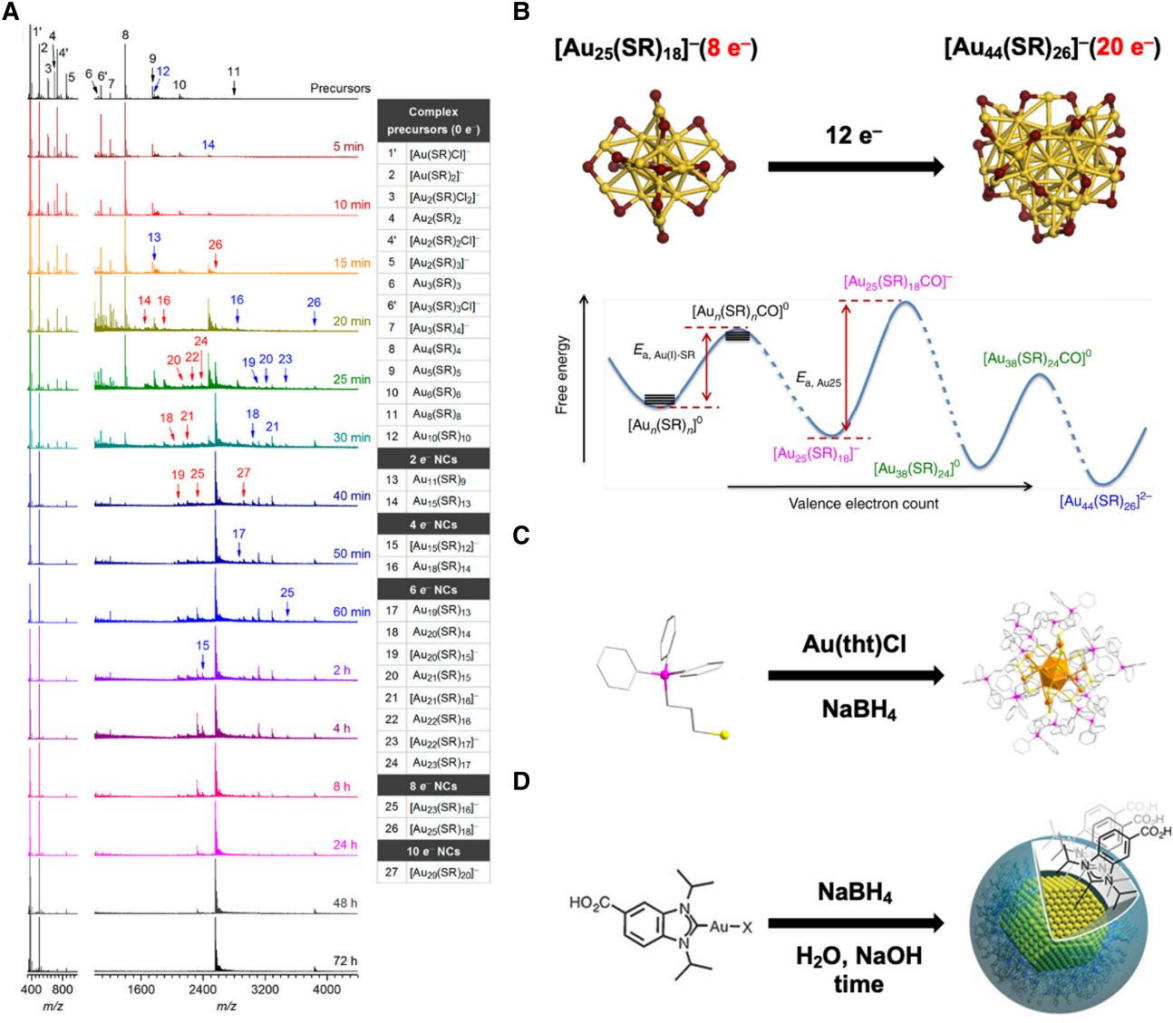
Illustration of bottom-up method of MNC synthesis. (A) Time evolution of electrospray ionization–MS spectra of the reaction solution during the synthesis of [Au_25_(SR)_18_]^−^. Reprinted with permission from Luo et al. (37). (B) CO-mediated transformation of Au_25_ NCs into Au_44_ NCs. Reprinted with permission from Yao et al. (38). (C) Water-soluble atomically precise Au_25_ NCs. Reprinted with permission from Hua et al. (39). (D) Synthesis of water-soluble NHC-protected Au NPs. Reprinted with permission from Salorinne et al. (40).

Bottom-up synthesis is widely used for MNCs and can be traced to the Brust-Schiffrin method (36). In a typical protocol for thiolate-protected Au NCs, Au(III) salts are reduced by thiols (SR) to form Au(I)–SR precursors (Au(III) + 3H–SR → Au(I)–SR + RS–SR + 3H^+^), followed by further reduction and growth. Building on the Brust-Schiffrin method, various refinements have enabled more controlled and insightful synthesis of atomically precise MNCs. Using CO as a mild reductant affords synthesis of monodisperse MNCs and permits in situ monitoring (UV-vis, MS), which has revealed a stepwise 2-electron growth pathway toward [Au_25_(SR)_18_]^–^ (Fig. 2A) (37). Tuning pH further adjusts the effective reducing ability of CO for precise control of sizes of MNCs (41). Similarly, using NaOH to moderate reducing ability, reducing agent NaBH_4_ enables the stoichiometric synthesis of [Au_25_(*p*MBA)_18_]^–^ (*p*MBA = *para*-mercaptobenzoic acid) (34). In addition to starting from metal salts, bottom-up growth can proceed from small NC seeds, for example, CO-mediated growth from [Au_25_(SR)_18_]^–^ to larger species [Au_44_(SR)_26_]^2–^ (Fig. 2B) (38). Although Au(III) salts such as HAuCl_4_ are commonly used precursors in aqueous-phase synthesis, water-soluble NCs can also be prepared from Au(I) complexes; for instance, deprotonated thiols functionalized with phosphonium groups act as charged ligands that enhance aqueous solubility via electrostatic interactions and strong hydration (Fig. 2C) (39).

Besides well-studied thiolate-protected MNCs, other ligands have also been used to expand the family of water-soluble MNCs. Protein-templated (42) and DNA-templated (43, 44) MNCs integrate biomolecules, offering intrinsic aqueous dispersibility together with prospects for bioimaging and therapy. For example, bovine serum albumin has been developed for the preparation of highly stable Au NCs with red emission and high quantum yield (42). *N*-heterocyclic carbenes (NHCs) diversify the ligand choices for water-soluble MNCs by introducing a distinct bonding motif: thiolates often bridge surface metal sites to form staple structures (40), whereas NHCs bind directly to core metal atoms (Fig. 2D) (45). These different interfacial interactions enable tunable solubility and open routes to new functions.

#### Top-down method

**Fig. 3.**
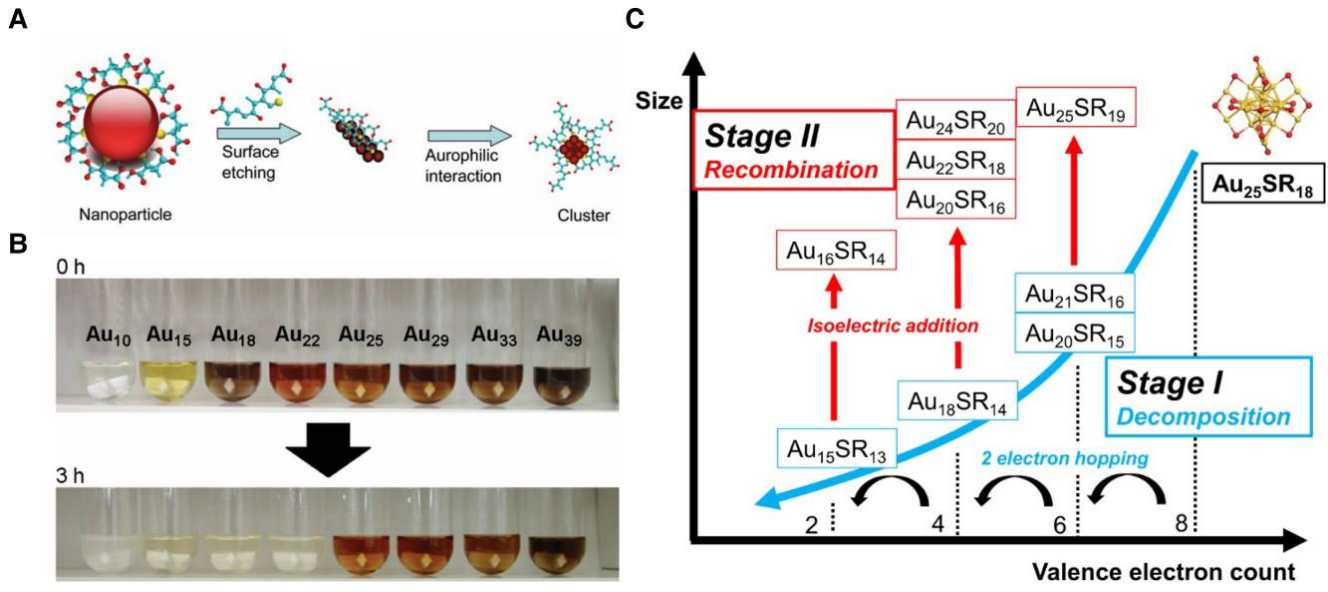
Illustration of the top-down method of MNC synthesis. (A) Schematic representation of the formation of Au_25_ NCs from Au@MSA NPs. Reprinted with permission from Habeeb Muhammed et al. (49). (B) Aqueous solutions of different Au_n_(SG)_m_ NCs before (0 h) and after (3 h) the reaction with GSH. Reprinted with permission from Shichibu et al. (50). (C) Two stages dominated by different reaction processes are shown. Stage I is decomposition and stage II is recombination. Reprinted with permission from Cao et al. (51).

Top-down synthesis of MNCs is governed by oxidative etching, which selectively converts larger NPs or NCs to smaller, thermodynamically favored species (46). Etching provides an effective route to synthesize monodisperse NCs, either by downsizing larger NPs or by refining polydisperse mixtures (47, 48). A representative early example is the conversion of 4 to 5 nm mercaptosuccinic acid–protected Au NPs into fluorescent Au_25_ NC or Au_8_ NC by etching with excess glutathione (GSH/SG), with the product selection governed by pH (Fig. 3A) (49). Etching also refines polydisperse larger mixture of Au_n_(SG)_m_ NCs by excess free GSH ligands selectively to Au_25_(SG)_18_ and Au(I) complexes—the 2 most stable products—enabling large-scale synthesis of Au_25_ NCs (Fig. 3B) (50).

Long-term in situ MS has deepened the mechanistic understanding of etching reactions. In the case of Au_25_(SR)_18_, the process involves 2 distinct stages: (i) decomposition via reverse growth through 2-electron steps and (ii) recombination through isoelectric addition of small fragments and Au(I) complexes (Fig. 3C) (51). The controlled nature of etching also allows for the rational design and synthesis of other stable or compositionally unique clusters. For example, oxidative transformation of [Au_25_(MHA)_25_]^–^ (MHA = 6–mercaptohexanoic acid) can yield smaller, luminescent [Au_22_(MHA)_18_]^0^ clusters (52), or a precise single ligand control addition to [Au_25_(MHA)_19_]^0^ by the etching method (53).

### Postsynthetic modification

Postsynthetic modification improves water solubility through interfacial engineering of postsynthesized hydrophobic clusters. Compared with direct synthesis, which often faces challenges in crystallization and structural characterization, postmodification offers a route to transfer well-defined clusters synthesized in organic media into aqueous environments. One strategy involves modifying the MNC surface through ligand exchange reactions or the introduction of polar functional groups via ligand engineering, which helps retain the core-shell structure while maintaining the small size of the NCs. Another approach is to encapsulate hydrophobic clusters within hydrophilic matrices such as amphiphilic polymers or surfactants. While this method minimizes structural changes to the MNCs, the resulting products are typically large micelle-like materials containing multiple NCs per carrier.

#### Surface modification

Surface modification is a widely used strategy for tuning NC properties while preserving the original core-shell structure and ultrasmall sizes, thereby retaining their molecule-like characteristics. As essential components of NC structures, ligands not only stabilize the metal core, but also mediate interactions with the surrounding environment. Tailoring these ligands therefore provides an effective means to regulate interfacial interactions and enhance water solubility.

**Fig. 4.**
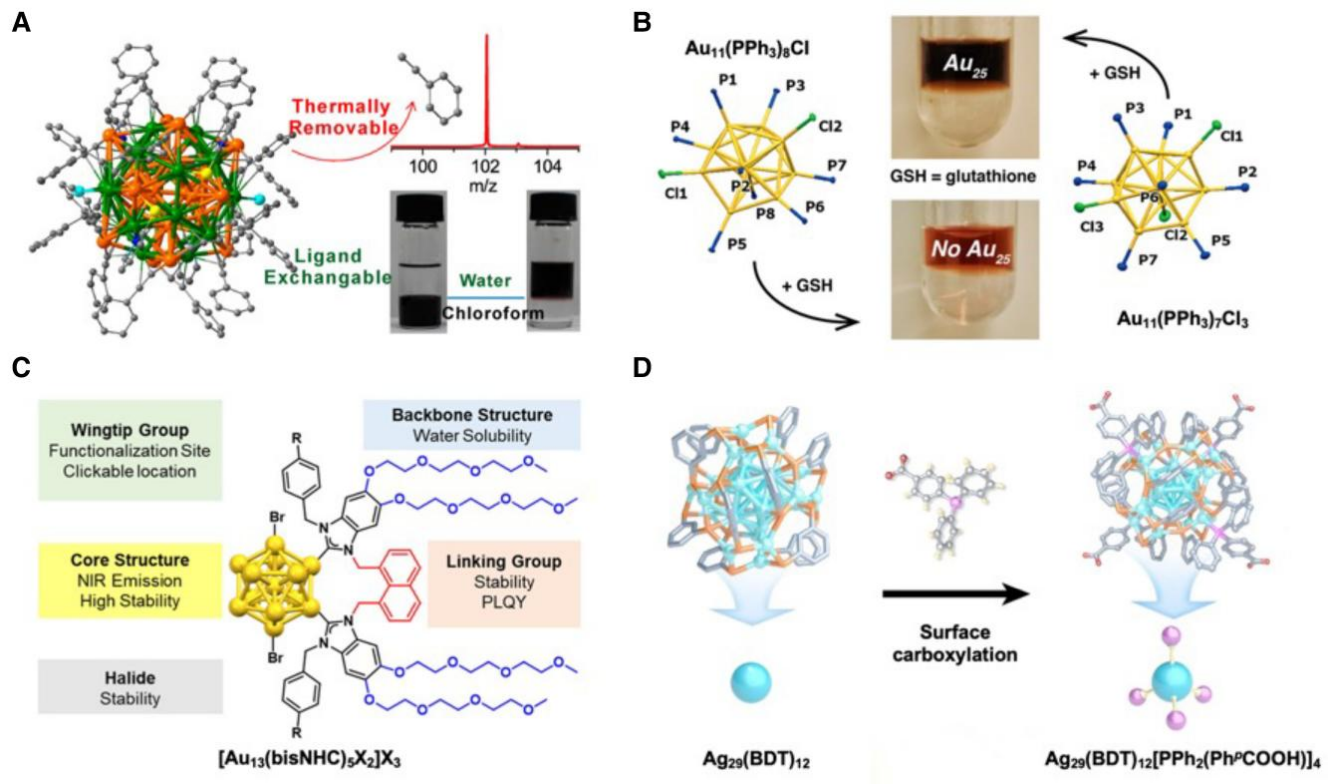
Surface modification of functionalizing MNCs. (A) Au_24_Ag_20_ NC transfer from organic phase to aqueous phase by ligand exchange. Reprinted with permission from Wang et al. (56). (B) Different exchange pathways of [Au_11_(PPh_3_)_8_Cl_2_]Cl and Au_11_(PPh_3_)_7_Cl_3_. Reprinted with permission from McKenzie et al. (57). (C) Illustration of surface modification of NHC-protected Au NCs designed for improving water solubility. Reprinted with permission from Sullivan et al. (58). (D) Illustration of surface carboxylation of Ag_29_(BDT)_12_ to Ag_29_(BDT)_12_[PPh_2_(Ph*^p^*COOH)]_4_. Reprinted with permission from Wei et al. (59).

Owing to the reversible and dynamic nature of metal-ligand bonds, ligand exchange can occur when incoming ligands temporarily coordinate to the surface and replace existing ones, allowing the system to reach a lower free energy state (54, 55). This process is influenced by thermodynamic, kinetic, and concentration factors. Ligand exchange can lead to two general outcomes. In isostructural exchanges, the metal core remains intact while surface ligands are replaced. For example, Au_24_Ag_20_(2-SPy)_4_(PhC ≡ C)_20_Cl_2_ shows nearly identical UV-vis absorption features after four 2-SPy ligands and 2 Cl^–^ anions are replaced by carboxyl-functionalized thiolate ligands (SPyCOOH), enabling the NCs to transfer from the organic phase to aqueous phase while retaining their structure (Fig. 4A) (56). Similarly, ligand exchange with water-soluble mercaptosuccinic acid has also been demonstrated for [Ag_141_Br_12_(S-Adm)_40_]^3+^, providing a representative example of hydrophilic modification (60). In contrast, some exchanges leads core rearrangements and induce size and structural transformations, as in the case of Au_11_(PPh_3_)_8_Cl_3_ converting to more stable Au_25_(SG)_18_ in the presence of GSH (61). Notably, even clusters with a similar composition can exhibit different exchange pathways depending on the stability of their ligand shells, as exemplified by [Au_11_(PPh_3_)_8_Cl_2_]Cl and Au_11_(PPh_3_)_7_Cl_3_ (Fig. 4B) (57). At the same time, at the length scale of atomically precise clusters, surface-targeted reactions may be coupled to atom exchange or kernel rearrangement, so ligand exchange can occasionally result in ligand-induced core modification rather than a purely interfacial process.

In addition to ligand exchange, another strategy is to functionalize the existing ligand scaffold in order to regulate interfacial interactions with the surrounding aqueous environment while maintaining the kernel. For example, introducing triethylene glycol functional groups onto ligands of NHC-protected MNCs imparts water solubility because of the introduction of hydrogen-bond networks (Fig. 4C) (58). Similarly, carboxylation of PPh_3_ ligands in Ag_29_(BDT)_12_(PPh_3_)_4_ enhances water solubility because of electrostatic interactions while retaining π-π interactions critical for ordered assembly (Fig. 4D) (59).

#### Encapsulation strategy

Encapsulation, widely used for stabilizing polymer-coated quantum dots (62, 63) and formulating NP drug carriers (64), has been extended to hydrophobic MNCs to achieve aqueous dispersibility while preserving their atomic precision. This allows structurally well-defined MNCs, previously synthesized in organic phases, to function in aqueous phase environments relevant to catalysis and biomedicine (65).

**Fig. 5.**
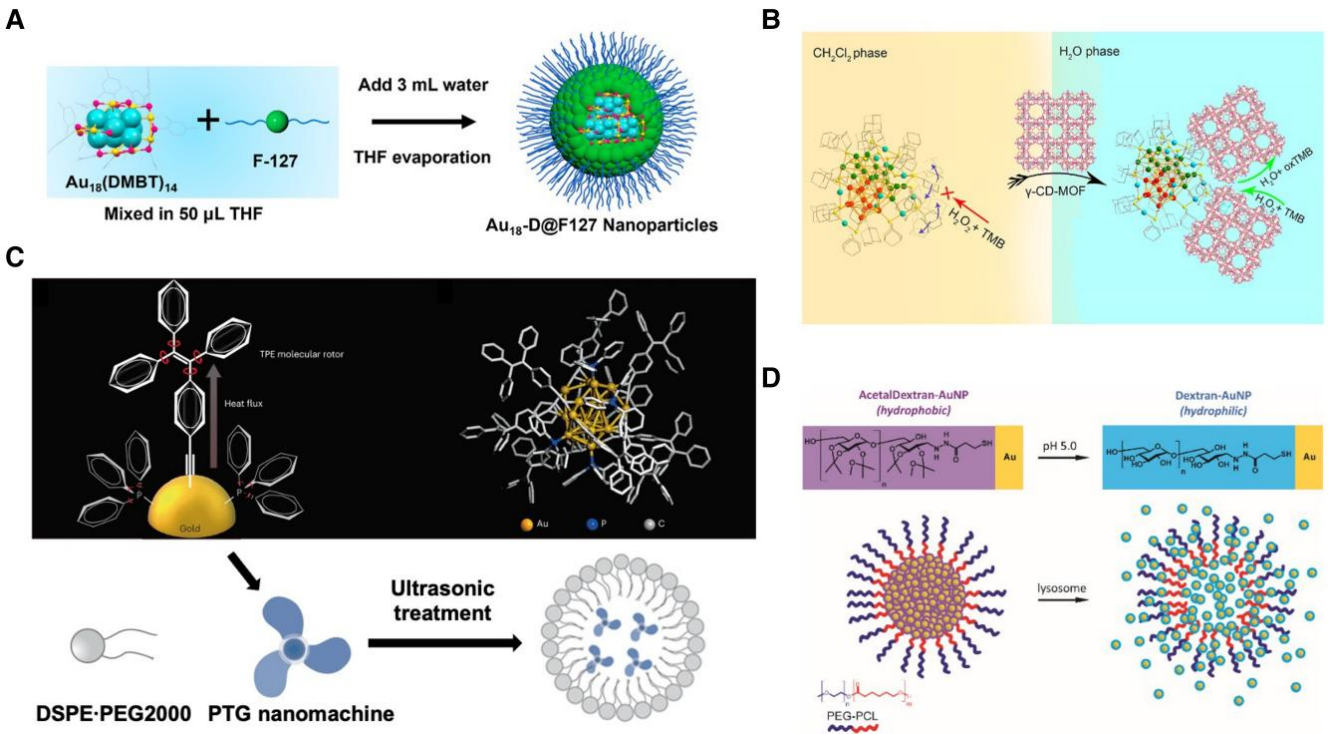
Encapsulation strategy of functionalizing MNCs. (A) Schematic formation process of Au_18_-D@F127 NPs. Reprinted with permission from Casteleiro et al. (66). (B) Schematic of phase transfer of Au_40_(S-Adm)_22_ enabled by host-guest chemistry incorporated into γ-cyclodextrin-based MOFs. Reprinted with permission from Zhao et al. (67). (C) Schematic illustration of PTG nanomachine encapsulation by DSPE-PEG2000 under ultrasonic treatment, with the nanomachine structures shown above. Reprinted with permission from Chen et al. (68). (D) Schematic of AcetalDextran-Au NPs: micelles remain stable at physiological pH but dissociate in acidic environments, releasing dispersed nanoparticles. Reprinted with permission from Higbee-Dempsey et al. (69).

Encapsulation enhances solubility and stability without disturbing hydrophobic ligand scaffolds. For example, Au_18_(DMBT)_14_ (DMBT = 2,4-dimethylbenzenethiolate) is transferred to water using amphiphilic Pluronic F127. The resulting clusters retain π-π and C–H···π interactions from the aromatic ligands, improving antioxidant stability and modestly enhancing photoluminescence (PL) by suppressing vibrational quenching (Fig. 5A) (66). Likewise, Au_40_(S-Adm)_22_ has been successfully incorporated into γ-cyclodextrin (CD)–based metal-organic frameworks (MOFs) through host-guest recognition (Fig. 5B). This process introduces a rigid hydrophilic outer layer while leaving the hydrophobic ligand scaffold intact, leading to stable aqueous dispersion and catalytic activation (67).

Encapsulation also offers opportunities to enhance functionality beyond solubility. PEG-encapsulated [Au_20_(TPEC ≡ C)_8_(PPh_3_)_6_]^2+^ NCs form suprastructures that enhance electron-phonon coupling, increasing photothermal conversion efficiency for tumor ablation (Fig. 5C) (68). DNA-Ag_16_ NCs confined in liposomes exhibit brighter fluorescence due to local concentration, enabling enhanced signal intensity for imaging applications. This confinement also improves their stability and biocompatibility in complex biological environments (70). Additionally, Au NPs coated with pH-sensitive acetylated dextran can be packed into polymeric micelles, where acidic disassembly exposes hydrophilic particles that degrade more readily, imparting stimuli-responsive clearance and improved biosafety (Fig. 5D) (69).

## Interfacing interactions

Interfacial interactions critically influence the structural dynamics and electronic behavior of MNCs. Surface ligands can form hydrogen bonds and electrostatic interactions with surrounding solvents, counterions, neighboring NCs, or extended frameworks, enabling dynamic exchange and environmental responsiveness. These interactions not only govern the dispersion and stability of MNCs in solution, but also facilitate charge transfer, structural reorganization, and functional integration. Depending on the nature of the surrounding environment, such interactions can direct NCs assembly into ordered superstructures by balancing interparticle attraction and repulsion or modulate intracluster charge distribution through coupling with external electron acceptors or donors. These interfacial effects offer powerful levers to control performance in applications ranging from catalysis to sensing and optoelectronics.

### Interfacial assembly

The formation of ordered MNCs superstructures, whether through crystallization or self-assembly, is fundamentally governed by interfacial interactions. Achieving precise arrangement while maintaining dispersion stability requires a delicate balance between attractive and repulsive forces. In aqueous environments, however, dominant electrostatic repulsion and solvation effects present substantial challenges for ordered organization.

**Fig. 6.**
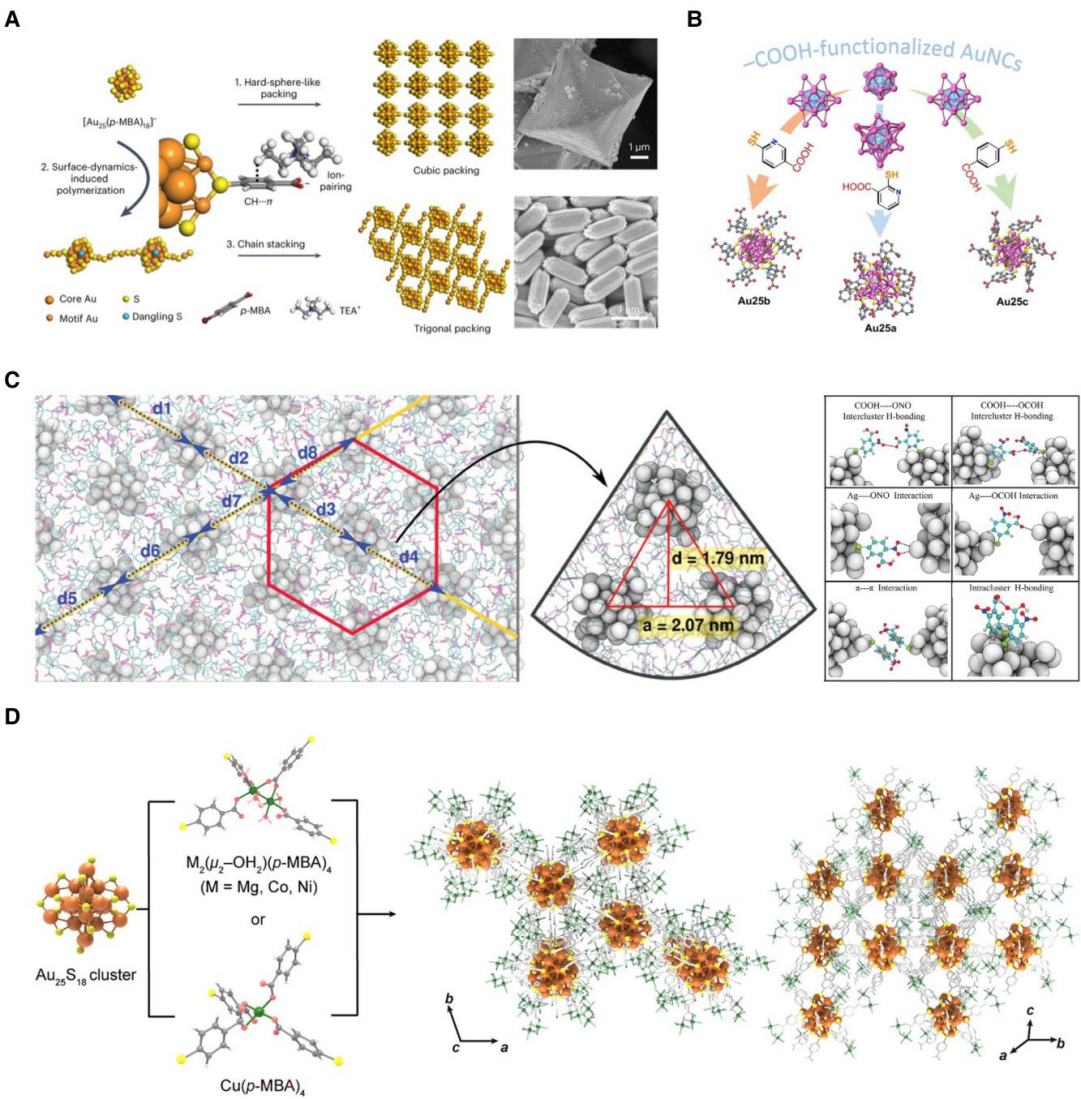
Functionalizing MNCs in interfacial assembly. (A) Schematic illustration of the crystallization of hard-sphere-like [Au_25_(*p*MBA)_18_]^−^ NCs into either fcc superlattices and octahedral crystals (top) or, in the presence of excess TEA^+^, an R-3m superlattice and hexagonal rod-like supercrystals (bottom). Reprinted with permission from Yao et al. (72). (B) Single-crystal structures of Au_25_ NCs protected by 3 types of –COOH-functionalized thiolate ligands. Reprinted with permission from Tian et al. (73). (C) Hexagonal close-packed structure of the Ag_44_(MNBA)_30_ NCs (H–bonds are shown in magenta color) and all possible ligand-mediated interactions. Reprinted with permission from Roy et al. (74). (D) Single-crystal structures of Au_25_ NC-based coordination frameworks, showing MOF-like assemblies in which different coordination metals (Mg, Co, Ni, Cu) regulate intercluster distances. Reprinted with permission from Kim et al. (75).

To address this, interfacial regulation strategies have been developed to guide NC assembly by tuning local environments. One approach involves charge compensation via counterions. For instance, cetyltrimethylammonium cations (CTA^+^) partially pair with deprotonated –COO^–^ groups on MHA-protected NCs, forming bilayer assemblies with regular interlayer spacing (71). Similarly, [Au_25_(*p*MBA)_18_]^–^ NCs assemble into long-range structures when paired with tetraethylammonium (TEA^+^), in which combined C–H···π and electrostatic interactions support structural cohesion. Low-dose transmission electron microscopy has visualized these assemblies, capturing bridging motifs between adjacent clusters (Fig. 6A) (72). Beyond ion pairing, hydrogen bonding plays a pivotal role in mediating aqueous phase assembly. In [Au_25_(*p*MBA)_18_]^–^, partial protonation via dialysis induces nanoribbon formation (76). Ag_44_(MNBA)_30_ exhibits a pH-dependent transition from dispersed clusters to hexagonally packed assemblies driven by the formation of extended hydrogen bonding networks (Fig. 6C) (74). Controlling surface interactions is crucial for directing ordered crystallization. –COOH groups, frequently present in water-soluble ligands, participate in electrostatic attraction, and also engage in π-π stacking, and coordination with metal ions. Given their weak ionization, carboxylates introduce charge heterogeneity during crystallization, hindering long-range ordering. Salting-out methods have been used to suppress electrostatic repulsion and weaken cluster-solvent interactions, allowing cluster-cluster interactions to dominate under supersaturated conditions, thereby facilitating single-crystal formation (Fig. 6B) (73). Alternatively, [Au_25_(*p*MBA)_18_]^–^ can be integrated into MOF-like frameworks through coordination with Zn^2+^ (77). The combination of metal-ligand bonding and hydrogen bonding among adjacent –COOH groups enables tailored packing modes and emission properties. Substituting Zn^2+^ with other cations (e.g. Mg^2+^, Co^2+^, Ni^2+^) further tunes intercluster distances and charge-transfer pathways, offering structural and functional flexibility (Fig. 6D) (75).

### Surface engineering

Rational surface design at the molecular level offers a versatile approach to modulate interfacial interactions between MNCs and their surrounding environment, enabling tunable optical properties and even structural transformations. By engineering surface chemistry, subtle forces such as hydrogen bonding, electrostatic interactions, and π-π stacking can be manipulated to influence emission behavior, structural dynamics, and even charge distribution at the atomic scale. In aqueous environments, these noncovalent forces act together with ligand-metal charge delocalization, in which partial electron sharing across the ligand-metal interface both stabilizes the core-shell framework and modulates the frontier orbitals that govern radiative versus nonradiative decay (78). Accordingly, tailoring the donor-acceptor character and conjugation of surface ligands provides a simple handle to jointly tune structural stability and luminescence in water (79).

**Fig. 7.**
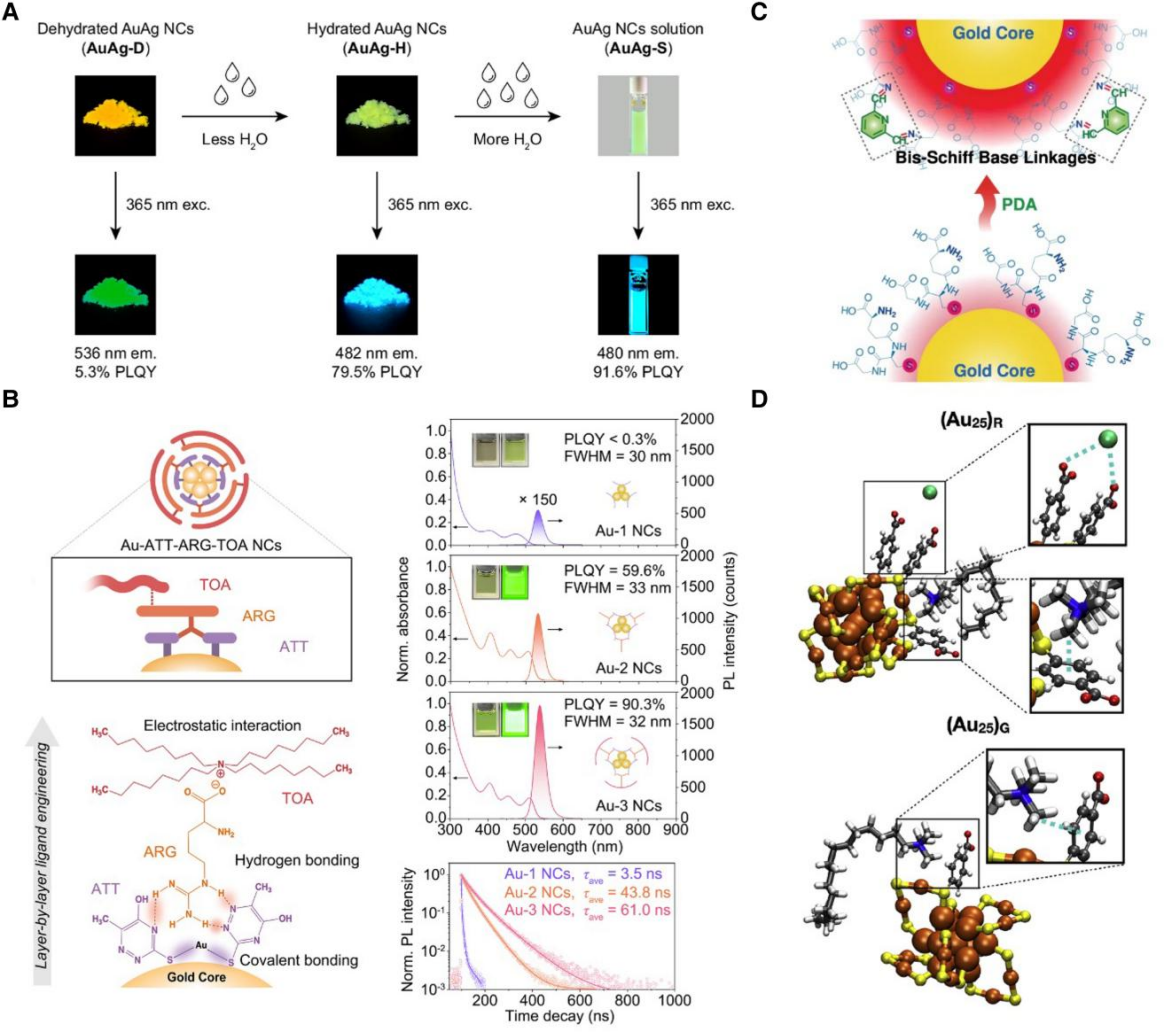
Functionalizing MNCs in surface engineering. (A) Water adsorption on AuAg NCs passivates defects, enhancing PLQY from 5.3% to 91.6%. Reprinted with permission from Zhong et al. (83). (B) Schematic illustration of layer-by-layer self-assembly of Au NCs with ATT, ARG, and TOA ligands, together with their optical and luminescence properties. Reprinted with permission from Zhong et al. (84). (C) Au_22_(SG)_18_ clusters exhibit enhanced emission via pyridine dicarboxaldehyde and *bis*Schiff base ligand crosslinking. Reprinted with permission from Deng et al. (85). (D) [Au_25_(*p*MBA)_18_]^–^ core structure is modulated by CTA^+^ interactions. Reprinted with permission from Cao et al. (86).

Solvent molecules are among the most ubiquitous regulators of interfacial behavior. In poor solvents, reduced solubility induces cluster aggregation and triggers aggregation-induced emission, which has been widely employed to boost the emission of intrinsically weakly luminescent MNCs (80). Beyond solvent-driven aggregation, cluster–ligand complexes and assembled states also exhibit aggregation-induced emission, as exemplified by Au(I)–thiolate systems in which Au/ligand stoichiometry and cation-induced aggregation switch between distinct emissive states (81) and mixed-valence Au(0)/Au(I) nanoclusters that reach high photoluminescence quantum yield (PLQY) white emission by tuning core-staple charge transfer (82). Moreover, subtle interactions between water molecules and NC surfaces can also dramatically alter photophysical behavior. For instance, water adsorption onto AuAg NCs passivates surface defects and blocks nonradiative pathways, shifting the emission to a fast core-based radiative route and enhancing the PLQY from 5.3 to 91.6% (Fig. 7A) (83). Beyond solvent effects, various external additives can be deliberately introduced to fine-tune interfacial forces. Cationic tetraoctylammonium (TOA^+^) ions adsorb electrostatically onto Au_22_(SG)_18_ surfaces, raising the PLQY from 8% to 62% (87). Zwitterionic surfactants such as BS-12 improve the emission of [Au_25_(*p*MBA)_18]–_ by simultaneously inducing hydrogen bonding and hydrophobic confinement, which suppress intercluster coupling and increase shell rigidity (88). Small molecules like ATT can establish hydrogen-bonded networks that rigidify the ligand shell and enhance PL. Subsequent assembly with TOA^+^ via electrostatic interactions further boosts PLQY beyond 90%, demonstrating a synergistic enhancement (Fig. 7B) (84). In a related system, ATT-functionalized Au NCs also exhibit markedly improved electrochemiluminescence in aqueous solution (89). In addition to noncovalent interactions, covalent ligand modifications provide structural reinforcement. For example, *bis*Schiff base formation between pyridine dicarboxaldehyde and amine-functionalized Au_22_(SG)_18_ enhances surface rigidity, resulting in brighter emission and accelerated relaxation dynamics (Fig. 7C) (85). Moreover, surface interactions such as C–H···π coupling between CTA^+^ and *p*MBA ligands can reversibly alter the core structure of [Au_25_(*p*MBA)_18_]^–^, underscoring the profound structural impact of interfacial chemistry at the atomic scale (Fig. 7D) (86).

## Applications for water-soluble MNCs

### Biological application

Water-soluble MNCs have emerged as promising candidates for biomedical applications owing to their unique combination of properties. Their excellent biocompatibility (90), low toxicity (91), and strong photostability make them suitable for use in complex biological environments. These basic safety and durability characteristics provide a foundation on which size-dependent transport, bright emission, and surface functionalization can be leveraged for more advanced biomedical tasks. Their ultrasmall size facilitates cellular uptake, while enhanced luminescence allows for efficient and sensitive cell labeling. Additionally, the presence of functional ligands on their surface enables further conjugation, supporting their use as drug delivery carriers.

#### Bioimaging

Bioimaging demands probes with high brightness, stability, biocompatibility, and tunable emission profiles. Water-soluble MNCs have emerged as ideal candidates due to their strong PL, excellent photostability, large Stokes shifts, low toxicity, and versatile surface chemistry, which make them suitable for in vivo imaging (92–94).

**Fig. 8.**
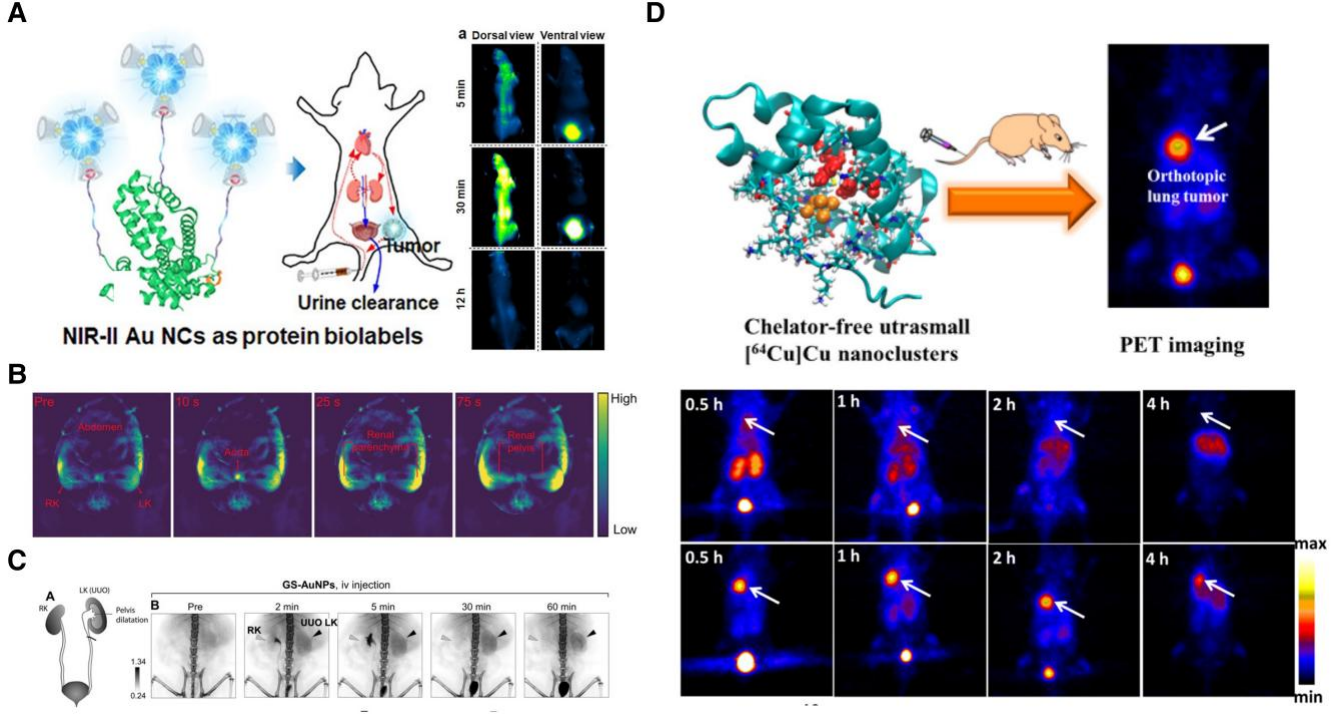
Functionalizing MNCs in bioimaging. (A) CD-protected Au NCs with host-guest supramolecular coatings provide stable NIR fluorescence imaging with efficient renal clearance and protein-specific targeting. Reprinted with permission from Song et al. (97). (B) Au_25_ NCs generate high-contrast photoacoustic signals for real-time visualization of renal transport. Reprinted with permission from Jiang et al. (98). (C) GSH-coated Au NCs act as radiosensitizers with good aqueous stability for noninvasive kidney function assessment via x-ray imaging. Reprinted with permission from Xu et al. (99). (D) 64Cu@BSA–Au NCs enable PET imaging for early and sensitive diagnosis of lung tumors. Reprinted with permission from Gao et al. (100).

Representative systems include DNA-stabilized Ag NCs (95), which have been used for selective membrane labeling of Chinese hamster ovary cells, and *bis*NHC-protected Au NCs (96), whose active uptake by human epithelial carcinoma cells has been confirmed via confocal microscopy. To achieve deeper tissue penetration, PL imaging in the near-infrared (NIR)-II window is especially desirable. Ligand and core engineering strategies have been employed to extend MNCs emission into the NIR-II range. For instance, CD-protected Au NCs not only enable stable NIR emission, but also exhibit efficient renal clearance. Their macrocyclic ligands allow high-affinity protein labeling via host-guest interactions without disrupting biological function, providing a robust platform for targeted imaging (Fig. 8A) (97).

Beyond PL imaging, MNCs are also applicable in photoacoustic imaging, which offers greater depth and resolution than fluorescence alone. Au_25_ NCs produce high-contrast photoacoustic signals and have been used to visualize renal transport pathways in real time (Fig. 8B) (98). In x-ray imaging, where soft-tissue contrast is often limited, GSH-coated Au NCs serve as effective radiosensitizers, enabling noninvasive kidney function assessment through differential retention in damaged and healthy tissues (Fig. 8C) (99). Moreover, MNCs have been adapted for positron emission tomography (PET). Radioactive ^64^Cu@BSA–Au NCs were developed for PET imaging-guided early yet sensitive diagnosis of primary orthotopic lung tumors (Fig. 8D) (100).

#### Bio-therapy

**Fig. 9.**
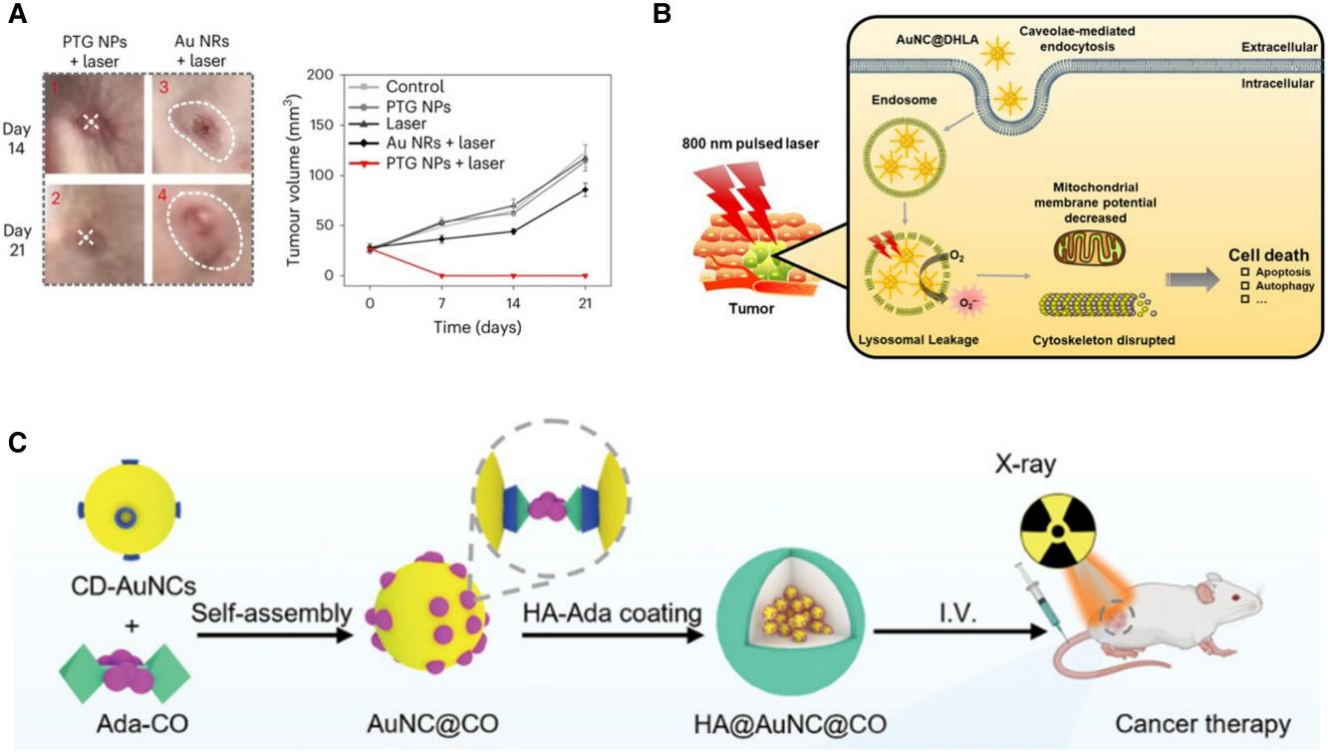
Functionalizing MNCs in biotherapy. (A) Enlarged tumor images and volume changes showing nonrecurrence in PTG+ laser-treated mice compared with Au nanorods. Reprinted with permission from Chen et al. (68). (B) DHLA-coated Au NCs generate ROS efficiently in lysosomes under 800 nm NIR light. Reprinted with permission from Han et al. (102). (C) HA@Au NCs@CO nanocomposites generate ROS and release CO under x-ray, inhibiting glycolysis and enhancing radiotherapy. Reprinted with permission from Cao et al. (103).

The therapeutic potential of nanomaterials is intrinsically linked to their decay pathways upon external excitation. Among these, light-induced excitation can lead to either radiative decay or nonradiative decay, the latter being harnessed for photothermal therapy. In photothermal therapy, materials with strong absorption convert photon energy into localized heat, inducing tumor apoptosis via protein denaturation, membrane rupture, and DNA damage. For instance, Au_44_(*p*MBA)_26_-Cy7 (Cy = heptamethine dye) combines Au NCs with a rigid photothermal dye, enhancing conversion efficiency (101). Similarly, [Au_20_(TPEC ≡ C)_8_(PPh_3_)_6_]^2+^ exhibits a photothermal conversion efficiency of ∼67.6% and retains structural integrity, achieving complete tumor ablation with a single laser dose in mice models while minimizing long-term toxicity (Fig. 9A) (68). In photodynamic therapy, MNCs generate reactive oxygen species (ROS) upon irradiation. DHLA-coated Au NCs, internalized into lysosomes via caveolae-mediated endocytosis, efficiently generate ROS under 800 nm NIR light (Fig. 9B) (102). Besides light-driven ROS generation, some nanomaterials can also trigger ROS production through chemical or catalytic pathways. For example, Ang-Te-Ag-Ce NPs use Ag^+^ catalytic sites to activate ROS production without light, promoting glioma cell death through oxidative stress (104).

Beyond light-based strategies, MNCs also enhance radiotherapy. For example, host-guest assembly of Adm-functionalized carbonyl compounds (Ada-CO) within CD-protected Au NCs yields HA@Au NCs@CO nanocomposites. Upon x-ray exposure, they produce ROS and simultaneously release CO gas, which inhibits glycolysis, synergistically boosting radiotherapy efficacy (Fig. 9C) (103). Additionally, Au NCs can be in situ biosynthesized within cancer cells by injecting Au(III), leveraging cancer cells’ innate advantage in Au(III) uptake and particle formation (105). These clusters sensitize tumors to x-ray–induced damage by disrupting DNA repair, inducing lipid peroxidation, and interfering with metabolism.

#### Enzyme-mimic catalysis

**Fig. 10.**
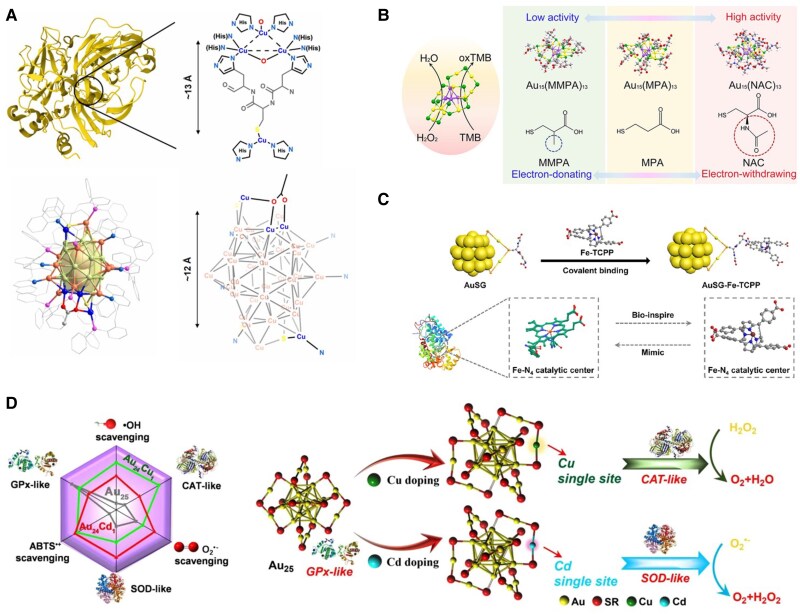
Functionalizing MNCs in enzyme-mimic catalysis. (A) Illustration of [Cu_36_L_7_(S-Adm)_3_(OAc)]^3+^ having similar structures to natural laccase. Reprinted with permission from Wang et al. (108). (B) Schematic representation of the peroxidase-mimic activity of Au_15_(SR)_13_ NCs protected by different ligands. Reprinted with permission from Shan et al. (109). (C) Illustration of the preparation process of AuSG-Fe-TCPP for mimicking the active center of natural peroxidase. Reprinted with permission from Fan et al. (110). (D) Schematic illustration of catalytic selectivity in the clusterzyme system: Au_25_ NC favors GSH peroxidase–like activity, Au_24_Cu_1_ NC shows catalase-like activity, and Au_24_Cd_1_ NC exhibits superoxide dismutase–like activity. Reprinted with permission from Liu et al. (111).

Natural enzymes are indispensable biological catalysts, yet their limited stability, high cost, and strong environmental dependence constrain widespread use (106). Due to their protein-like hierarchical structure, combined with the established methodology for customizing the property-determining attributes at the atomic level, MNCs have been demonstrated to exhibit catalytic activities resembling those of natural enzymes (107). This dual feature—structural analogy to proteins and atomically precise programmabilityunderpins their role as enzyme mimics. One representative strategy exploits the intrinsic similarity between MNCs surface motifs and enzymatic coordination environments. For example, [Cu_36_L_7_(S-Adm)_3_(OAc)]^3+^ (L = 2-(2-(diphenylphosphaneyl)phenethyl)quinoline) reproduces the multinuclear copper center of laccase (Fig. 10A) (108). The resulting cluster exhibits laccase-like oxidase activity together with higher catalytic rates, enhanced stability, and broader operational tolerance than the natural enzyme.

Building from analogy to programmability, kernel-level substitution offers direct control of electronic structure and reactivity. Replacing 1 Au atom in Au_22_(SG)_18_ with Cu to form Au_21_Cu_1_(SG)_18_ increases catalase-like activity by about 90-fold, consistent with a modest band-gap contraction that facilitates charge transfer and lowers barriers (112). Dopant identity also tailors selectivity: within the Au_25_ NC family, Au_25_ displays GSH peroxidase–like behavior, Au_24_Cu_1_ is biased toward catalase-like activity, whereas Au_24_Cd_1_ favors superoxide dismutase–like pathways, which illustrate how kernel composition programs reaction channel (Fig. 10D) (111). At the ligand and surface level, programmability further expands the enzyme-mimicking scope of MNCs. The Au_15_(SR)_13_ NC illustrates how ligand identity influences peroxidase-like activity, with density functional theory (DFT) analysis attributing the superior performance of Au_15_(NAC)_13_ to the electron-withdrawing effect of the acetylamino group (Fig. 10B) (109). Beyond ligand chemistry, partial removal of protecting ligands has been calculated to expose undercoordinated Au sites, thereby reshaping catalytic pathways and facilitating charge transfer (113). Surface modification offers yet another avenue: incorporation of Fe-TCPP (TCPP = *meso*-tetra(4-carboxyphenyl)porphine) onto GSH-protected Au NCs generates hybrid clusterzymes with enhanced affinity for H_2_O_2_ and accelerated electron transfer (Fig. 10C) (110), while conjugation with natural enzymes, as in Au NC-lysozyme-curcumin assemblies, combines cluster activity with protein-derived stability and biocompatibility (114).

#### Electrocatalytic reactions

In electrocatalysis, aqueous electrolytes define both the medium for ion transport and the environment in which interfacial charge transfer occurs. Their high dielectric constant, safety, and low cost make them suitable for application-relevant operation. Water-soluble, atomically precise MNCs can capitalize on this environment: hydrophilic ligand shells confer strong affinity for the aqueous phase, which can create preferred microenvironment for efficient adsorption and diffusion of small substrates.

**Fig. 11.**
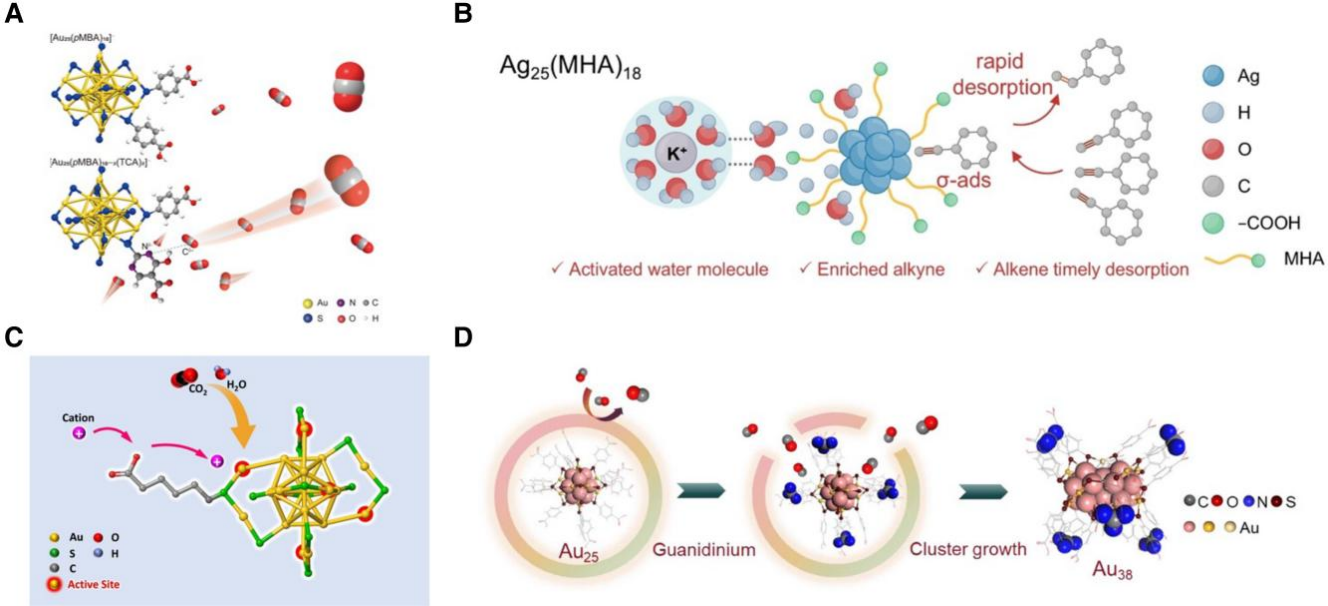
Functionalizing MNCs in electrocatalysis. (A) TCA ligands enhance the CO_2_ enrichment efficiency on Au_25_ NCs. Reprinted with permission from Liu et al. (116). (B) Ag_25_(MHA)_18_ catalyze alkyne semihydrogenation by colocalizing water and alkyne at the interface. Reprinted with permission from Zhang et al. (117). (C) Schematic illustration of electrocatalytic CO_2_ reduction reaction taking place on a Au_25_ NCs protected by cation-relaying ligands. Reprinted with permission from Han et al. (118). (D) Guanidinium cations reorganize the ligand shell of *p*MBA-protected Au NCs, opening transport pathways and enhancing CO_2_ access. Reprinted with permission from Zhu et al. (119). I.V., intravenous.

Intrinsic ligand chemistry tunes interfacial electronics and recognition. On [Au_25_(SR)_18_]^−^, comparing thiolates of similar size—*p*MBA, MHA, and homocysteinate—reveals that stronger electron withdrawal increases the partial positive charge on Au(I) within Au(I)–SR motifs, strengthens OH^−^ chemisorption in alkaline media, lowers intermediate barriers, and shifts the rate-determining step of the oxygen evolution pathway (115). The same logic extends to recognition motifs: precisely positioning 2 TCA ligands inside the pocket of [Au_25_(*p*MBA)_18_]^−^ creates a CO_2_ affinity site; supramolecular interactions between the nucleophilic pyrimidinyl nitrogen and the electrophilic carbon of CO_2_ enrich the reactant near the active atom and accelerate CO_2_ reduction kinetics (Fig. 11A) (116). Ligand-layer organization steers adsorption geometry and product release. In electrochemical semihydrogenation of alkynes, Ag_25_(MHA)_18_ leverages the amphiphilicity and ordered packing of MHA to colocalize water and alkyne at the interface; the enzyme-like pocket favors σ-bond binding of terminal alkynes to Ag sites and expedites olefin desorption, suppressing overhydrogenation (Fig. 11B) (117). Cation-relay ligands represent another design strategy. Replacing neutral HT with MHA introduces carboxylate termini capable of coupling with electrolyte cations. Comparative studies showed that Au_25_(MHA)_18_ outperforms Au_25_(HT)_18_ in CO_2_-to-CO conversion once the electrolyte pH exceeds the pKa of MHA. Activities scale as Li^+^ < Na^+^ < K^+^ < Cs^+^, and Nernstian shifts with cation concentration confirm a cation-coupled electron transfer step governs the reaction (Fig. 11C) (118). Stimulus-driven supramolecular reorganization reprograms the shell without breaking Au–S connectivity. Guanidinium cations form ion pairs with carboxylates and build hydrogen-bond networks on *p*MBA-protected Au NCs, reorganizing the shell into micelle-like domains that open transport corridors to noncoordinated metal sites; CO_2_ access increases and CO_2_ reduction performance improves while the kernel remains intact (Fig. 11D) (119).

## Conclusion

Water-soluble MNCs have matured from curiosities at the molecule-nanomaterial boundary into programmable platforms whose functions arise from a tight coupling between atomic structure, ligand motifs, and aqueous interfacial chemistry. Direct aqueous synthesis—clarified by in situ MS and tandem MS—now resolve growth, reconstruction, and single-ligand events with molecular precision, while top-down etching provides complementary, shell-closure-guided routes to benchmark species. In parallel, postsynthetic strategies translate high-performance organic-phase clusters into water without sacrificing core identity, offering practical handles to tune dispersibility, stability, and biointerface while preserving intrinsic structure-property correlations.

A key insight emerging from this review is that “water solubility” is not merely a formulation constraint but an enabling design principle. Hydration, ion pairing, hydrogen bonding, and host-guest effects collectively script 2 powerful modes of interface control: interfacial assembly that organizes discrete NCs into cooperative superstructures and surface engineering that creates ligand-defined pockets and charge/proton relays. These aqueous interactions rigidify shells to suppress nonradiative loss, bias adsorption equilibria, and gate proton-coupled electron transfer, thereby linking supramolecular environment to photophysics and catalytic kinetics.

These advances translate into versatile applications. In biomedicine, renal-clearable, NIR-emissive clusters support high-contrast imaging and image-guided therapy, while redox- and photothermal-active systems deliver therapeutic efficacy with mechanistic traceability. In catalysis, atomically defined cores wrapped by programmable ligand shells function as enzyme-like active sites in water, where pocket geometry, local dielectric, and ion/proton relays overcome diffusion limits and control selectivity. In electrocatalysis, hydration-coupled modulation of Au(I)–SR motifs and ligand-directed substrate enrichment provide levers to raise activity and shift rate-determining steps under operando aqueous conditions.

Looking forward, several priorities could accelerate translation from mechanistic insight to deployable function: (i) quantitative descriptors that connect ligand-shell rigidity, hydration structure, and interfacial dielectric to rates, selectivity, and PL (120); (ii) data-centric discovery, spanning sequence-defined templating and machine-learning-guided design, to navigate large ligand/composition spaces (121); (iii) scalable, batch-consistent syntheses and robust processing into films/electrodes that retain molecular precision (122, 123); and (iv) standardized assessments of pharmacokinetics, immunocompatibility, and long-term fate to bridge laboratory function with clinical or environmental use (124). By integrating atomic-level control with aqueous interfacial programming, water-soluble MNCs provide a rare opportunity to design the microenvironment as carefully as the active site. This convergence positions the field to deliver mechanistically transparent imaging agents, therapeutics, and catalysts in which performance, stability, and safety are co-optimized from the molecule upward.

## Data Availability

This study did not generate new data. All data discussed in this review are available in the cited literature.
